# Talent Selection Strategies and Relationship With Success in European Basketball National Team Programs

**DOI:** 10.3389/fpsyg.2021.666839

**Published:** 2021-06-11

**Authors:** Anton Kalén, Alexis Padrón-Cabo, Erik Lundkvist, Ezequiel Rey, Alexandra Pérez-Ferreirós

**Affiliations:** ^1^Faculty of Education and Sport Sciences, University of Vigo, Pontevedra, Spain; ^2^Department of Psychology, Umeå University, Umeå, Sweden; ^3^Unit of Investigation in Human Nutrition, Growth and Development of Galicia (GALINUT), University of Santiago de Compostela, Santiago de Compostela, Spain

**Keywords:** talent identification, team sport, sport federation, national sporting organizations, youth national team, countries

## Abstract

There is limited knowledge of the talent selection strategies used by national sporting organizations to identify and develop talented players in basketball. Therefore, we aimed to explore differences in selection strategies between European youth basketball national team (NT) programs, and how they relate to the program’s success. Specifically, we examined differences in the number of youth NT players and within-country variance in the 1988–1999 generations between 38 countries (*n* men = 38, women = 32). Further, we tested if the number of youth NT players and within-country variance was related to the NTs senior ranking, youth ranking, and youth-to-senior player promotion, using generalized Bayesian multilevel models. We further checked the moderating effect of the amount of licensed basketball players in each country. On average, 15.6 ± 2.0 male and 12.4 ± 1.8 female players were selected per generation. Over a third of the NTs consistently selected a higher or lower number of players than the average, with a difference of 8.1 players (95% CI [5.8, 10.8]) for men and 7.6 players (95% CI [5.4, 10.0]) for women between the countries with the highest and lowest average. When licensed players were used as moderator, the differences decreased but did not disappear, in both genders. There was an above 99.3% probability that a higher number of players was positively related to higher men’s senior and youth rankings, and women’s youth ranking. Within countries, generations with a higher number of youth players generated more senior players, with a probability of 98.4% on the men’s, and 97.3% on the women’s side. When licensed players were used as moderator, the probabilities for these relationships remained largely unaffected, apart from women’s youth ranking, which sank to 80.5%. In conclusion, the selection strategy in basketball NT programs varies between European countries and selecting a higher number of players possibly relates to better long-term performance and more players promoted to the senior NTs. These findings show that talent development programs should make conscious decisions about their selection strategies as it can affect their success.

## Introduction

Nations, through sporting organizations, are making increasingly large investments to achieve sporting success (De Bosscher et al., 2015). An important part is the investment in talent development and pathways to increase the performance at the senior level (De Bosscher and De Rycke, 2017; De Bosscher et al., 2018). In team sports, especially in Europe, one of the main talent development programs organized by the national federations are the youth national team (NT) programs, which aim at developing better senior NT players.

These talent development programs can adopt different strategies aiming to reach their ultimate goal. Previous literature has, for example, contrasted two different strategies (Güllich and Emrich, 2012; Barth et al., 2018). The first – an individualistic approach – is characterized by an early selection of players followed by a long-term development of this particular group resulting in a low turnover of players. The second – a collectivistic approach – is characterized by selecting and de-selecting players throughout the pathway with a higher turnover of players (Güllich and Emrich, 2012; Barth et al., 2018). Earlier research has shown that talent development programs in team sports are generally characterized by a collectivistic approach. In German Olympic sports NT programs, for example, only around half of the involved players remained selected from one season to the next (Güllich and Emrich, 2012). In team sport youth NTs, between a third and a half of the players are de-selected from one season to the next (Barreiros et al., 2012; Güllich, 2014; Wrang et al., 2018).

However, a recent study found that European youth basketball NT programs have a lower turnover of players, with 70–80% re-selected from 1 year to the next (Kalén et al., 2020). Furthermore, another study showed that European senior basketball NT players had played an average of around three youth championships (Kalén et al., 2017). These findings suggest that basketball youth NT programs might use more of an individualistic approach. While these findings suggest a more individualistic approach, no previous study has directly analyzed the characteristics of European youth NT selection strategies. Although several studies have investigated selection strategies in a variety of sports (Güllich, 2014; Barth et al., 2018; Bjørndal et al., 2018), there is little knowledge of how much the selection strategies differ between different programs or countries within the same sport.

While the individualistic and collectivistic approaches are built on different ideas of how talent identification and selection should be made, both aim at maximizing international success by promoting the athletes from the youth program with the best possibility of high performance at the senior level. This allows us to compare the success of different selection strategies. Earlier basketball research has, for example, found a relationship between higher rates of re-selection in youth NT programs and better long-term performance of the senior NT (Kalén et al., 2020). A possible explanation discussed is that higher re-selection rates might be an indication of better organization and clearer strategies within the program, which has been shown to lead to better performance (De Bosscher et al., 2015). It is, therefore, possible that there exists an association between countries’ selection strategies and their performance over time.

Further, as the idea behind both more individualistic and more collectivistic approaches is that senior success is created by promoting athletes from the youth program, the selection strategies aim to maximize the number of youth players who reach the senior NT. That is, the number of senior NT players that were selected in the youth NTs is another measure of how successful different selection strategies are. An earlier study has found that players who participated in European senior basketball championships had, on average, played 2.3 and 3.2 youth championships, for men and women, respectively (Kalén et al., 2017). It was also found that better-performing men’s teams had a higher number of accumulated youth championships in the rosters (Kalén et al., 2017). It is therefore likely that having players with youth NT experience can help the team’s performance. The proportion of youth NT players who reach the senior NT seem to vary significantly between sports and countries. For example, in Portugal, over 50% of youth male volleyball NT players, but only around 35% of the male football players reach the senior NT (Barreiros and Fonseca, 2012). Meanwhile, in German man’s football, it has been reported that only 5% of players make the transition from youth to the senior NT (Schroepf and Lames, 2018). While this difference probably can be explained in part by differences in study methodology, it does suggest that there may be differences between different countries in the proportion of players that do reach senior NTs.

One challenge in researching talent selection strategies is to understand to what degree they reflect conscious strategical decisions made by the programs, and to what degree they are the result of factors outside the programs themselves. For example, aspects such as national sport policies, the financial and sporting strength of clubs, the professional status of youth coaches, and the popularity of the sport might very well play an important role in shaping the selection strategies used. It is, however, generally difficult to find reliable measures comparing multiple countries for most of these factors. One measure that is attainable across European countries is the number of licensed players. Although the link between sport participation in a country and success is somewhat complicated (De Bosscher et al., 2015), investigating the moderating effect of the number of licensed players could give an initial indication of how much the strategies might be influenced by factors outside the programs.

Given the differences in selection strategies, both between sports and countries, more detailed studies are needed to better understand selection strategies used in different sports and countries. Further, given the scarce literature, it is of interest to explore how effective different strategies are at generating sporting success. The main purpose of the current study was, therefore, to explore talent strategies used in European basketball NT programs and how they might relate to the success of the NT programs. The specific aims were: (a) Examine if selection strategies for youth NTs differ between countries. (b) Examine if the selection strategy is associated with the success of the NT program. (c) Examine to what degree the number of licensed players in each country moderates the effects for aims (a) and (b). (d) We also aimed to evaluate the senior NT debut age and the proportion of senior NT players that have played in the youth NTs.

## Materials and Methods

**FIGURE 1:**
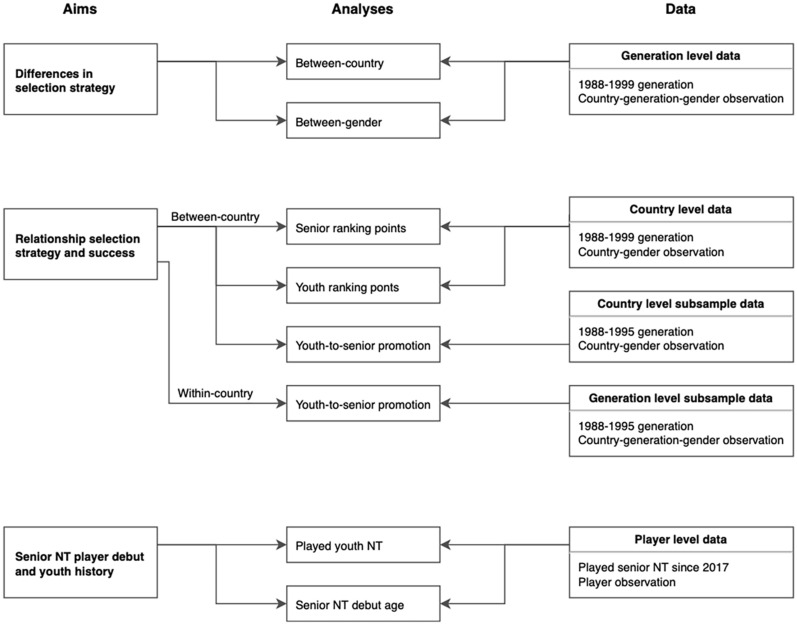
Flowchart of aims, analyses, and data of the study.

The current study consists of retrospective analyses of register data on participation in basketball championships for players from European countries. We used official data on players’ NT participations, NT rankings, and number of licensed players in the countries. The participation data was registered by the organizing committee of each competition in the official competition system used during the championships. The ranking points are calculated by the International Basketball Federation based on the official final game results and is used for seeding in future championships. The number of licensed players was self-reported by each national basketball federation. All data was obtained from the official website of the International Basketball Federation (FIBA; fiba.com). The analyses were performed separately for men and women. An overview of the study can be seen in Figure 1.

### Sample

For the analysis of differences in selection strategy and its effect on country ranking, the participation history of all players from the 1988 to 1999 generations, that had played at least one youth championship was used – corresponding to championships played 2004–2019. We refer to a generation as all players born in the same calendar year (i.e., players born 1988 comprise one generation, 1989 another, etc.). On the men’s side, 6619 players, from 38 countries and 408 country-generation combinations were included. On the women’s side, 4,433 players from 38 countries and 254 country-generation combinations were included. Only players from countries in which a minimum of five of the twelve generations participated in youth championships were included.

For the analysis of selection strategy effect on the number of youth NT players that reach the senior NT, we included only players born 1988–1995. This subsample consisted of 4,389 male players from 269 country-generation combinations and 2,932 female players from 232 country-generation combinations. We chose this subsample as all players born 1995 and earlier had reached age 25 in the last included season (2019). Using age 25 as a cut-off for having made senior debut allowed us to conclusively classify all included players as either having reached the senior NT or not, in line with earlier studies (Wrang et al., 2018). Furthermore, the senior debut analysis confirms that 89–90% of senior NT players that played youth NT had debuted at age 25 (see section 3.4), supporting the soundness of the cut-off.

For the analysis of the proportion of senior NT players with youth NT experience and their senior debut age, the participation history of all players who had participated in a senior NT championship since 2017 was used. On the men’s side, 348 players who had participated in the 2019 World Cup or 2017 Eurobasket were included. On the women’s side, 293 players who had participated in the 2019 Eurobasket, 2018 World Cup, or 2017 Eurobasket were included.

### Variables

To quantify the selection strategy, we first counted the number of players that had played at least one youth championship in each generation and country (Nr Youth Players). For each country, we then calculated the average number of youth NT players per generation, as well as the coefficient of variation (CV) between the generations. The CV was expressed as the percentage of the mean and calculated as 100 × country standard deviation/country mean.

Based on the goals of talent programs discussed in the introduction, we used three different measures of NT program success: (1) senior ranking points, which indicates how well the senior NT has performed over time; (2) youth ranking points, which indicates how well the youth NTs have performed over time; and (3) the number of youth NT players that reach the senior NT, indicating how successful the youth NT program is at promoting players to the senior NT (youth-to-senior NT promotion).

Both the senior and youth ranking points are calculated by FIBA based on the NT performance over the last eight seasons and are calculated separately for men and women. The senior ranking points are based on the results in the senior European and World Championships (Eurobasket and World Cup), as well as the respective qualifications. The youth ranking points are based on the performance in the annual U16, U18, and U20 European Youth Championships, as well as the biennial U17 and U19 World Youth Championships. The rankings were transformed to a 0–1 range, where 1 represents the maximum number of ranking points attainable. The ranking was further transformed using the formula (ranking × (*n* − 1) + 0.5)/*n*, as beta regressions cannot handle 0’s in the outcome variable (Smithson and Verkuilen, 2006).

To quantify the youth-to-senior promotion success, we counted the number of players in each of the 1988–1995 generations that had played at least one youth championship and one official game with the senior NT before or at the age of 25 (Nr Senior Players). For each country, we then calculated the average number of youth-to-senior NT players per generation.

We used the reported number of licensed basketball players for each gender in the country as a potential moderating variable on the relationship between programs’ strategy and success. Visual inspection of the data revealed the number of licensed players per country to be approximately log normal. It was, therefore, transformed using a natural logarithmic transformation to avoid problem with skewness.

For the analysis of the proportion of senior NT players with youth NT experience and their senior debut age, we classified players as “played youth” or “senior only”, depending on if they had participated in at least one youth championship or not. The players’ senior debut age was calculated by subtracting their birth year from the year in which they played their first official senior NT game (championship or championship qualification).

### Analysis

To analyze the difference in selection strategy between countries, as well as the general difference between genders, we fitted a Bayesian linear multilevel model (Gelman and Hill, 2007), with nr youth players as the outcome, using generation level data. The model included a gender effect, and generations were nested within country. The posterior predictive distribution of specific countries’ means and CV of nr youth players were compared to the overall mean and CV within each gender to determine which countries had a diverging selection strategy diverging. CV was computed by dividing the estimated standard deviation by the estimated mean within each posterior draw. Further, the posterior predictive distribution of the difference between the country with the highest and lowest number of players, as well as between genders were estimated. A moderator model was fitted by updating the original model with a separate effect for the number of licensed players within each gender. This allowed us to test to what degree potential differences in selection strategies are driven by the number of licensed players in each country.

To analyze the effect of selection strategy on the senior and youth ranking points, we fitted a multivariate Bayesian beta regression (Smithson and Verkuilen, 2006), with the senior and youth ranking points of each country as outcomes, using country-level data. We used a beta-regression as the ranking points have a lower and upper bound, restricting the potential values. The model included an effect for gender, as well as separate effects for mean and CV of nr youth players within each gender. A moderator model was fitted by updating the original model with an effect for the number of licensed players within each gender. This allowed us to test to what degree the relationship between selection strategy and country ranking is explained by the number of licensed players in each country. We present the posterior predictive distribution of the relationship visually, together with the observed values for each country.

We analyzed the effect of selection strategy on youth-to-senior NT promotion success in two ways: a between-country analysis (in line with the ranking points analysis), and a within-country analysis of how nr youth players in each generation affect the nr senior players from that generation. For the first one, we fitted a Bayesian linear regression (Gelman and Hill, 2007), with the standardized mean nr senior players as the outcome, using country-level data. The model included an effect for gender, as well as separate effects mean and CV of nr youth players within each gender. For the second, we fitted a Bayesian Poisson multilevel regression (Gelman and Hill, 2007), with nr senior players as the outcome, using generation level data. We used a Poisson regression as nr senior players is a count variable. Generations were nested within country, and the model included an effect for gender, as well as a separate effect for nr youth players for each gender. For both models, a moderator model was fitted by updating the original model with effects for the number of licensed players and senior ranking points within each gender. This allowed us to test to what degree the relationship between selection strategy and nr senior players is explained by the number of licensed players in each country and the countries long-term performance. We present the posterior predictive distribution of the relationship visually, together with the observed values for each country in the country-level analysis, and posterior prediction for each country in the within-country analysis.

All analyses were performed in R 4.0.3 and models were fitted in STAN using the brms package (Bürkner, 2017). As we did not possess strong prior subject knowledge, we used weakly informative priors in all models to only provide slight regularization and, therefore, avoid overconfidence in the results. The models were estimated using a Hamiltonian Monte Carlo algorithm with four chains, each with 5,000 warm-up and 5,000 sampling iterations. No models showed any divergent transitions and the maximal $\hat{\text{R}}$ value was 1.0015 across all models (Betancourt and Girolami, 2015). Models were compared with their respective moderator model using the relative approximate leave-one-out cross-validation (ELDP loo), where the model with the value further from zero show worst predictive performance (Vehtari et al., 2017). Compatibility intervals (CI) were calculated using the highest-density intervals. The interpretation of, for example, the 95% CI is that there is a 95% probability that the value falls within this range. Furthermore, the probability of direction (PD) was calculated for the effects. The PD provides an estimate between 0.5 and 1 indicating the probability of the effect being positive for positive effects and negative for negative effects. For example, PD = 0.5 indicates an equal probability that the effect is positive or negative, while PD > 0.999 indicates that the effect is almost certain (Makowski et al., 2019). The datasets analyzed and the software for this study can be found in the Open Science Framework repository https://doi.org/10.17605/OSF.IO/ZHVDS.

## Results

Descriptive statistics for each country can be found in Supplementary Tables 1, 2, for men and women, respectively.

### Selection Strategy

**FIGURE 2:**
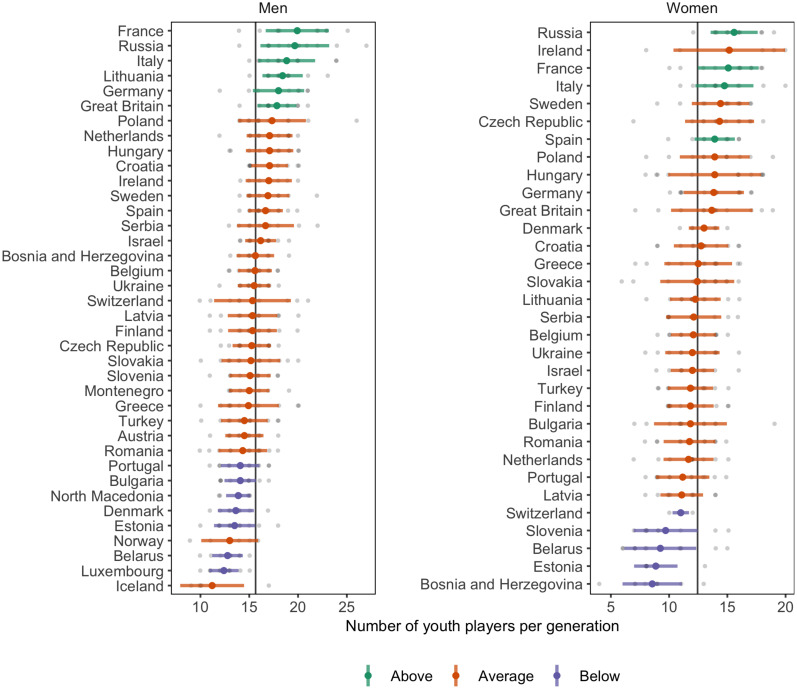
Average number of youth players per generation for each country, with colors indicating differences from overall average.

**FIGURE 3:**
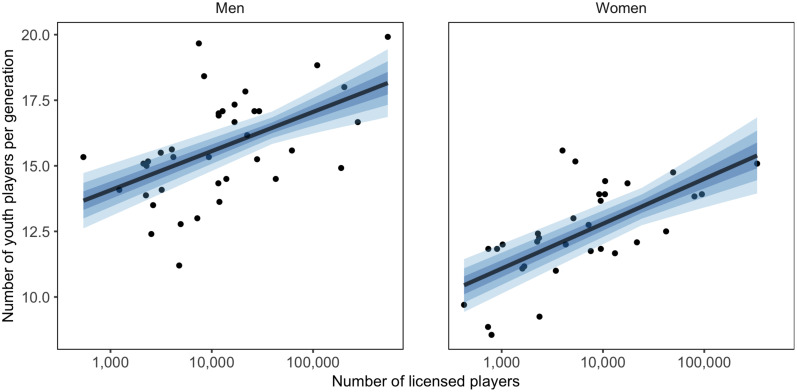
Relationship between countries’ reported number of licensed players (on log scale) and mean number of youth players per generation, with 50, 80, and 95% CI of posterior predicted distribution.

The average number (and standard deviation) of players that had played youth championship per generation is presented for each country and gender in Figure 2. To test for the potential influence of the number of basketball players in each country, a multilevel regression model with the number of licensed players as moderator was used. The moderator model showed marginally better predictive performance (ELDP diff = −4.1, SE = 2.8). The posterior predicted distribution and observed values of the relationship between the number of licensed players, on a log scale, and the average number of players per generation is shown in Figure 3.

On the men’s side, countries had an average of 15.6 ± 2.0 players per generation that had played youth championships, ranging from 11.2 to 19.9 players. The average CV for the number of players between generations within each country was 15.0 ± 4.3%, ranging from 9.0 to 29.2%. The median number of reported licensed male players per country was 11,651 (IQR = 3,411–25,360), ranging from 540 to 546,632.

Six countries had a higher, and seven a lower-than-average number of players per generation (PD > 0.950), indicated by color in Figure 2. When the number of licensed players was used as moderator, two countries had a higher, and two had a lower-than-average number of players per generation. The estimated difference between the highest and lowest countries was 8.1 players (95% CI [5.8, 10.8]), and in the moderation model for the number of licensed players 6.9 players (95% CI [4.6, 9.5]). No country differs from the overall average CV. In the moderation model one country showed a lower-than-average CV.

On the women’s side, countries had an average of 12.4 ± 1.8 players per generation that had played youth championships, ranging from 8.6 to 15.6 players. The average CV for number of players between generations within each country was 20.1 ± 6.1%, ranging from 6.4 to 33.3%. The median number of reported licensed female players per country was 5,221 (IQR = 2,099–11,158), ranging from 425 to 332,555.

Five countries had a higher, and five a lower-than-average number of players per generation (PD > 0.950), indicated by color in Figure 2. When the number of licensed players was used as moderator, one country had a higher-than-average number of players per generation, and no country a lower. The estimated difference between the highest and lowest countries was 7.6 players (95% CI [5.4, 10.0]), and in the moderation model for the number of licensed players 6.2 players (95% CI [4.1, 8.6]). Two countries had a below-average CV, with no change in the moderation models.

There was an average of 3.2 more male than female players per generation, 95% CI [2.7, 3.8]. The within-country variation was 5.1 percentage points lower in men compared to women, 95% CI [1.9, 8.3 points]. When the number of licensed players was used as moderator, the difference in the average number of players diminished to 2.9, 95% CI [2.2, 3.5], and the difference in within-country variation remained largely unaffected (4.7, 95% CI [1.3, 8.0]).

### Selection Strategy and Country Ranking

**FIGURE 4:**
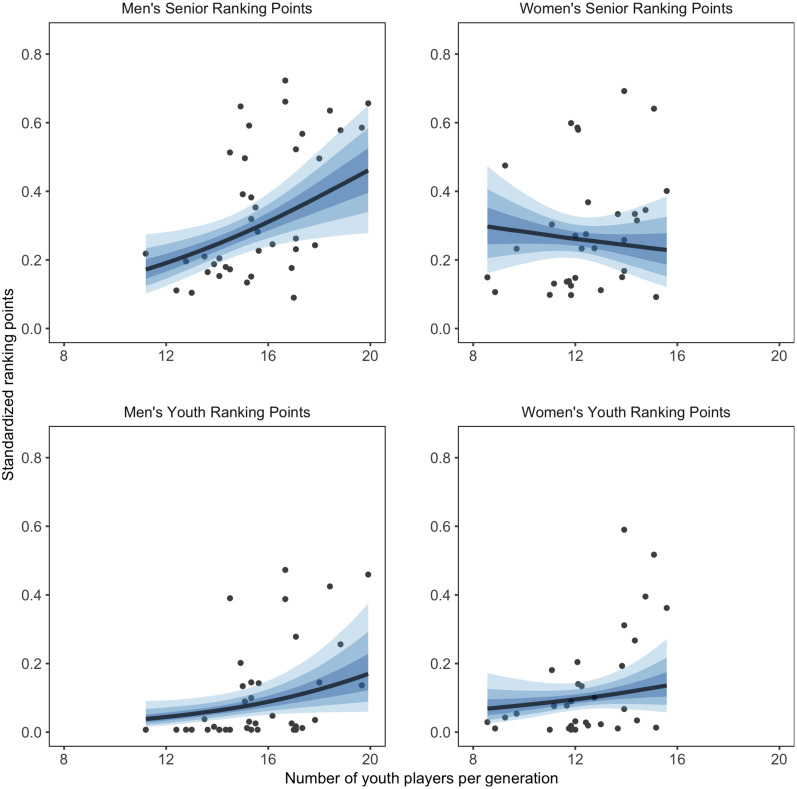
Relationship between countries’ mean number of youth players per generation and ranking points controlled for number of licensed players, with 50, 80, and 95% CI of posterior predicted distribution.

Result for the relationship between countries’ selection and ranking points, together with the moderating effect of nr licensed players is shown in Table 1. The moderator model did not show better predictive performance (ELDP diff = −5.0, SE = 5.8). The relationship between the average number of youth players per generation and ranking points is shown in Figure 4.

**TABLE 1:** Multivariate Beta-regression estimates for influence of countries’ mean and CV of youth players per generation on youth and senior ranking points.

	Model			Moderator model		
		
	Est	95% CI	PD	Est	95% CI	PD
**Senior ranking**						
Men						
Intercept	−0.98	[−1.30, −0.65]	>0.999	−1.09	[−1.43, −0.77]	>0.999
Mean nr youth players	0.58	[0.27, 0.86]	>0.999	0.41	[0.05, 0.75]	0.989
CV nr youth players	0.06	[−0.27, 0.39]	0.646	0.04	[−0.28, 0.35]	0.603
Nr licensed players				0.38	[−0.03, 0.76]	0.969
Phi	2.06	[1.60, 2.49]		2.15	[1.69, 2.59]	
Women						
Gender	0.28	[−0.23, 0.78]	0.859	−0.06	[−0.68, 0.55]	0.577
Mean nr youth players	0.27	[−0.15, 0.65]	0.907	−0.13	[−0.64, 0.34]	0.706
CV nr youth players	−0.04	[−0.31, 0.22]	0.624	0.03	[−0.23, 0.27]	0.579
Nr licensed players				0.63	[0.12, 1.13]	0.992
Phi	1.90	[1.41, 2.38]		2.08	[1.58, 2.56]	
**Youth ranking**						
Men						
Intercept	−2.55	[−3.10, −2.01]	>0.999	−2.67	[−3.26, −2.11]	>0.999
Mean nr youth players	0.61	[0.21, 1.02]	0.998	0.46	[−0.02, 0.95]	0.968
CV nr youth players	−0.05	[−0.48, 0.35]	0.589	−0.06	[−0.47, 0.35]	0.599
Nr licensed players				0.34	[−0.25, 0.94]	0.869
Phi	1.77	[1.25, 2.27]		1.82	[1.30, 2.34]	
*Women*						
Gender	1.08	[0.36, 1.78]	0.999	0.66	[−0.19, 1.48]	0.940
Mean nr youth players	0.64	[0.13, 1.16]	0.993	0.27	[−0.36, 0.86]	0.805
CV nr youth players	−0.17	[−0.47, 0.12]	0.873	−0.13	[−0.41, 0.17]	0.800
Nr licensed players				0.77	[0.20, 1.34]	0.994
Phi	1.83	[1.27, 2.35]		2.09	[1.50, 2.64]	
**Relative model performance**						
ELDP diff (SE)	−5.0 (5.8)			0		

*CV: coefficient of variation; ELDP diff: relative approximate leave-one-out cross-validation; SE: standard error; CI: compatibility interval; PD: probability of direction.*

On the men’s side, a higher number of youth players per generation positively related to both senior and youth ranking (PD > 0.999 and PD = 0.998). When the number of licensed players was used as moderator the positive relations held (PD = 0.989 for senior; PD = 0.968 for youth). On the women’s side, a higher number of youth players per generation positively related to both senior and youth ranking with a probability of 0.907 and 0.993, respectively. However, when licensed players were used as moderator the positive relations became weaker (PD = 0.706 for senior; PD = 0.805 for youth).

There was a 0.873 probability that a higher CV related to lower youth ranking points on the women’s side. When the number of licensed players was used as moderator this relation decreased (PD = 0.800). There was a < 0.650 probability of relationships between the CV and the men’s and women’s senior ranking, as well as men’s youth ranking, both with and without including moderation effect for the number of licensed players.

### Selection Strategy and Youth-to-Senior NT Promotion

**FIGURE 5:**
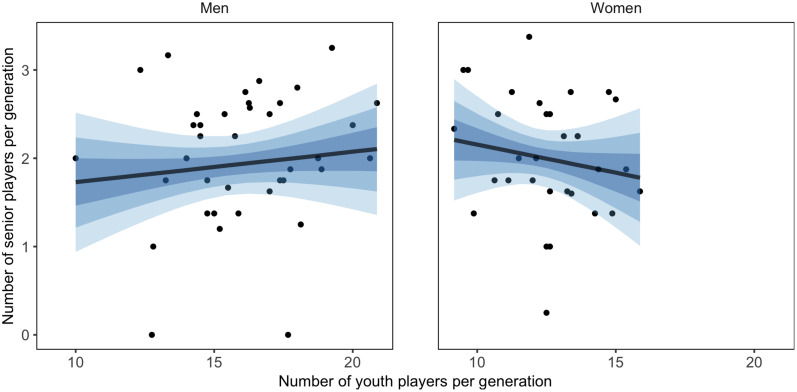
Relationship between countries’ average number of youth players per generation and the number of players that reach senior controlled for number of licensed players, with 50, 80, and 95% CI of posterior predicted distribution.

Results for the relationship between countries’ selection strategies and the youth-to-senior promotion, together with the moderating effect of nr licensed players and senior ranking is shown in Table 2. The moderator model showed a considerably better predictive performance (ELDP diff = −165.3, SE = 4.3). The relationship between the average number of youth players per generation and amount of youth players that had debuted with the senior NT at age 25 per generation is shown in Figure 5.

**TABLE 2:** Linear regression estimates for influence of countries’ mean and CV of youth players per generation on mean number of youth players that reach senior.

	Model			Moderator Model		
		
	Est	95% CI	PD	Est	95% CI	PD
Men						
Intercept	1.89	[1.58, 2.21]	>0.999	1.86	[1.35, 2.38]	>0.999
Mean nr youth players	0.13	[−0.14, 0.40]	0.833	0.09	[−0.26, 0.44]	0.706
CV nr youth players	−0.14	[−0.46, 0.19]	0.800	−0.13	[−0.48, 0.19]	0.776
Nr licensed players				0.06	[−0.37, 0.48]	0.618
Senior ranking				0.06	[−1.47, 1.55]	0.530
Women						
Gender	−0.03	[−0.53, 0.49]	0.544	0.21	[−0.60, 1.05]	0.690
Mean nr youth players	−0.20	[−0.63, 0.21]	0.833	−0.17	[−0.71, 0.37]	0.738
CV nr youth players	0.07	[−0.19, 0.32]	0.705	0.07	[−0.20, 0.34]	0.712
Nr licensed players				0.03	[−0.53, 0.56]	0.551
Senior ranking				−0.70	[−2.34, 0.95]	0.804
Residuals						
Sigma	0.74	[0.61, 0.87]		0.75	[0.62, 0.89]	
Relative model performance						
ELDP diff (SE)	−165.3 (4.3)			0		

*CV: coefficient of variation; ELDP diff: relative approximate leave-one-out cross-validation; SE: standard error; CI: compatibility interval; PD: probability of direction.*

On the men’s side, a higher number of youth players per generation was possibly related to a higher number of players reaching the senior NT (PD = 0.833). However, when licensed players and senior ranking were used as moderators, the probability of a positive relationship decreased (PD = 0.706). On the women’s side, a higher number of youth players was possibly related to a lower number of players reaching the senior NT (PD = 0.833). However, when licensed players and senior ranking were used as moderators, the probability of a negative relationship decreased (PD = 0.738). The probability of the relationship between the CV and the number of players reaching the senior NT was ≤ 0.800 for both genders, both with and without including moderation effect for the number of licensed players and senior ranking.

**FIGURE 6:**
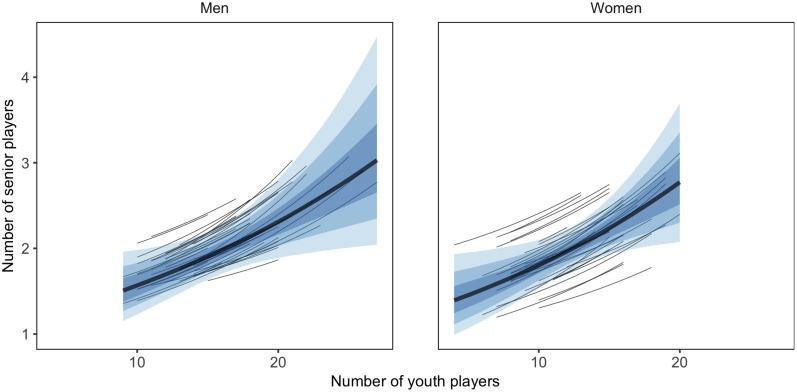
Within-country relationship between number of youth players and number of players that reach senior in a generation controlled for number of licensed players and senior ranking, with 50, 80, and 95% CI of posterior predicted distribution. Lines indicate posterior prediction for each country.

Results for the within-country relationship between a generation’s number of youth players and the youth-to-senior promotion, together with the moderating effect of nr licensed players and senior ranking is shown in Table 3. The moderator model showed a considerably better predictive performance (ELDP diff = −1,368.1, SE = 19.6). The relationship between a generation’s number of youth players and the number of them that had debuted with the senior NT at age 25 is shown in Figure 6.

**TABLE 3:** Multilevel Poisson regression estimates for within-country influence of number of youth players on number of players that reach senior per generation.

	Model			Moderator Model		
		
	Est	95% CI	PD	Est	95% CI	PD
Men						
Intercept	0.64	[0.52, 0.77]	>0.999	0.73	[0.50, 0.95]	>0.999
Nr youth players	0.12	[0.01, 0.22]	0.984	0.13	[0.02, 0.24]	0.989
Nr licensed players				0.02	[−0.16, 0.20]	0.602
Senior ranking				−0.32	[−0.98, 0.34]	0.836
Women						
Gender	0.10	[−0.07, 0.28]	0.878	0.13	[−0.19, 0.46]	0.792
Nr youth players	0.12	[0.00, 0.23]	0.973	0.15	[0.02, 0.27]	0.992
Nr licensed players				−0.11	[−0.29, 0.09]	0.865
Senior ranking				−0.30	[−1.03, 0.45]	0.791
Random effects						
Sigma intercept	0.17	[0.02, 0.29]		0.17	[0.02, 0.29]	
Sigma nr youth players	0.08	[0.00, 0.18]		0.08	[0.00, 0.18]	
Relative model performance						
ELDP diff	−1368.1 (19.6)			0		

*CV: coefficient of variation; ELDP diff: relative approximate leave-one-out cross-validation; SE: standard error; CI: compatibility interval; PD: probability of direction.*

On both the men’s and women’s side, a higher number of youth players in a generation was related to a higher number of the youth players reaching the senior NT for both men and women (men PD = 0.984; women PD = 0.973). When licensed players and senior ranking were used as moderators, the probability of a positive relationship increased (men PD = 0.989; women PD = 0.992).

### Senior Debut

**FIGURE 7:**
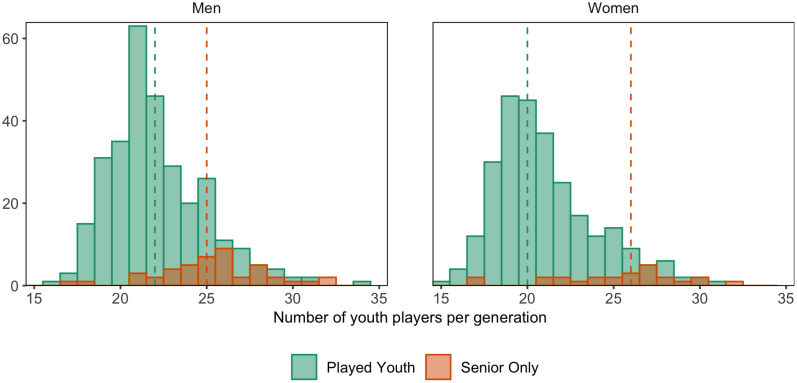
Distribution of senior national team (NT) debut age, by gender and if player had played in youth NTs or not. Dashed line indicates median debut age.

In the last two men’s senior championships, 303 of the 348 (87%) participating players had played in the youth NTs. The median senior debut age was 22 years (IQR = 20–24 years) for players who had played in youth NTs, and 25 years (IQL = 24–27 years) for players without youth NT experience. Of the players that had played in youth NTs, 269 (89%) had debuted at age 25. The distribution of debut age is shown in Figure 7.

In the last three women’s senior championships, 268 of the 293 (91%) participating players had played in the youth NTs. The median senior debut age was 20 years (IQR = 19–23 years) for players who had played in youth NTs, and 26 years (IQL = 23–27 years) for players who only had played in the senior NT. Of the players that had played in youth NTs, 243 (91%) had debuted at age 25. The distribution of debut age is shown in Figure 7.

## Discussion

In this study, we aimed to explore differences in selection strategies between youth basketball NT programs of European countries, and how they relate to the programs’ success. The main findings of this study were: (a) countries select an average of 15.6 males and 12.4 female players per generation, with individual generations differing 15.4% from the male, and 20.1% from female average; (b) countries differed considerably between each other in the average number of youth players selected per generation, but with great similarities in amount of variations between generations; (c) higher number of youth players per generation was related with a better youth and senior NT ranking in both genders; (d) there was no clear relationship between the within-country variation and NT ranking; (e) within countries, generations with a higher number of selected youth players had a higher youth-to senior NT promotion; and (f) the number of licensed basketball players in each country explain part of, but not all of the difference in selection strategies and its effect on the performance.

The talent identification process has been widely studied in individual and team sports (Johnston et al., 2018; Till and Baker, 2020; Williams et al., 2020). Specifically, basketball studies concluded that motor abilities (Erčulj et al., 2010) and maturational status (te Wierike et al., 2015; Arede et al., 2019b, 2020) play an important role in players’ selection process and the progress of their careers. The present study revealed that the average number of players who participated in youth championships per generation was around 16 players in the men’s category and 12 in the female’s category. As the NT consists of 12 players per tournament, the generally most used strategy seems to be leaning toward being more individualistic than in earlier studies in other sports (Güllich and Emrich, 2012; Barth et al., 2018). Similar differences between sports have been found when studying the number of players re-selected from 1 year to the next, where the re-selection was much higher in European NT basketball than in both German and Portuguese football (Barreiros and Fonseca, 2012; Güllich, 2014; Kalén et al., 2020). These observed differences between sports could probably be related to the sport’s popularity within specific countries, which might increase the number of licensed players in these sports and, consequently, the number of players available to be selected.

When comparing selection strategies between countries, we found a considerable amount of variation in the average amount of players selected per generation. When controlling for the effect of number of licensed players, the variation decreased but did not disappear. This suggests that although some of the differences in the number of players selected seem to be explained by the number of players the NTs have available to select from, there are still considerable differences in selection strategies. This is, to our knowledge, the first study of differences in selection strategies between countries in team sports. However, it is in line with De Bosscher and De Rycke (2017), that found differences in the type of support services given in talent development programs between different countries.

The main aim of investing resources in national talent development programs, such as the basketball youth NTs, is to increase the country’s international performance (De Bosscher et al., 2018). In the current study, we measured the NT programs’ success using the official FIBA ranking points, which are given to countries based on their results during the last 8 years. It, therefore, provides a measure of the country’s long-term success. Furthermore, the rationalization for investing resources in talent development programs is that it produces success by promoting the best athletes to the senior teams, either through long-term development of a small number of early selected athletes (individualistic approach) or through trying out a larger number of athletes by continuous selection and de-selection (Barth et al., 2018). Regardless of the approach adopted, one central goal of the youth NT programs is, therefore, to promote youth NT players to the senior NT. We measured how many players debuted with the senior NT in the different countries, as well as within-county differences between generations.

The results of the present study seem to, overall, support the idea that more collectivistic approaches show better long-term effects both when it came to team performance and promoting players to the senior NTs. This is in line with earlier findings that both individual coaches and talent programs do not seem to identify future successful senior athletes much better than chance (van Rens et al., 2015; Schorer et al., 2017), and that we have limited knowledge of how to effectively identify and develop talent (Johnston et al., 2018; Till and Baker, 2020). Earlier talent research has largely focused on identifying factors that influence the development toward becoming an elite player (Torres-Unda et al., 2013; Garcia-Gil et al., 2018; Arede et al., 2019a), but there is very limited evidence on the influence of organizations selection strategies.

A possible explanation for the lack of a clear relationship between selection strategy and long-term performance on the women’s side is the low number of players selected, and the small spread between countries in comparison with the men’s side. Further, considerably fewer countries participate in each championship on the women’s side, which affects the distribution of ranking points. Finally, the lack of relationship between the number of youth players and the number of players promoted to the senior NT on a country level could be expected, as each country has a more or less fixed number of spots on the senior NT roster. Our findings, therefore, seem to suggest that the proportion of senior NT players that are promoted from the youth NT program is relatively stable between countries. Given the small number of players who are involved in the NT programs, it would be of interest to study selection strategies and success including both NTs and clubs. For example, considering both reaching senior NT and becoming a professional athlete as outcomes.

Earlier studies have found that more efficiently structured and organized talent development programs have higher success (Gonçalves et al., 2011; De Bosscher et al., 2015). We used the amount of variation in the number of youth players between the different generations as a measure of the stability of the country’s selection strategy. While we found some differences in stability between countries, this measure did not influence either the NT ranking or the youth-to-senior NT promotion. It might, however, be questionable to what degree the variation in number of selected players per generation reflect the degree of structure and organization of the program. It could be possible that this measure is influenced by factors external to the program, such as the number of highly skilled players available in the specific generation, number of players missing championships due to injuries and similar (te Wierike et al., 2015; Arede et al., 2019b). More studies looking at the organizational stability of youth NT programs are therefore needed before we should draw any conclusions from these results.

We further analyzed the proportion of senior NT players that had previously played in the youth NTs and their senior NT debut age. Nine of ten senior NT players had played in the youth NTs, which is similar to earlier reports in Norwegian handball (Bjørndal et al., 2018). This is, however, higher than six and seven of ten in Portuguese volleyball and soccer, respectively (Barreiros and Fonseca, 2012). These differences are likely to be the result of differences both in culture between countries, and popularity of the different sports. Furthermore, players with youth NT experience generally debuted at an earlier age, and about 90% of youth NT experienced players had debuted at age 25.

While we found that countries differed considerably in the number of players selected for the youth NTs over 12 generations, we do not know if this is a result of conscious strategic decisions or unconscious consequence of the condition in which the selections are made. It has been found that team sport coaches primarily make player selection decisions based on instinct and “gut feeling” (Christensen, 2009; Roberts et al., 2019). This is an indication that the differences in selection strategies might emerge from, for example, cultural differences in coaches’ reasoning to a higher extent than decisions on an organizational level. Multiple ways of increasing talent selection efficacy have been proposed, with the common theme of making selection criteria more explicit and less reliant purely on coaches’ tacit knowledge and intuitions (Musculus and Lobinger, 2018; Sieghartsleitner et al., 2019; Johnston and Baker, 2020). Even though our results do not give conclusive evidence of what selection strategy is the best, they do suggest that the selection strategy potentially influences the possibility for long-term success. National sporting organizations should, therefore, make sure that their selection strategies are the result of conscious and explicit decisions.

It is unclear to what extent the selection strategies reflect conscious strategic decisions or result from factors outside the NT programs, such as club structures, quality of youth coaches, or national policies. It is also unclear to what extent the higher success of programs selecting a higher number of players is a result of the selection strategies used, and how much is explained by other factors. Therefore, further studies are needed to more thoroughly study how to explain actual strategies and their outcomes. For example, by studies on a club or regional level, using more holistic and deeper research methods, and longitudinal studies – potentially with interventions.

A limitation of the current study is that we only included the total number of licensed players in the country as a potential reason behind differences in selection strategy and its effect on success. While we were limited to this measure, as it was the only one available across all included countries, future studies should address the influence of more detailed influences. Examples of interesting measures to include in future studies are the number of available players at the specific moment of selection in different generations, the strength of clubs in the country, and the number of professional youth coaches in the country. As many of these measures are hard to reliably attain across all European countries, more holistic case studies, using interviews with coaches, review of strategy documents, and detailed information on surrounding influences would add valuable knowledge on the area. Further, the number of licensed players was self-reported by each national federation, and there might be discrepancies in how these values were obtained.

Another limitation is the summary view that the number of selected players (and re-selection proportion) gives of selection strategy. For example, having a core of early, long-term involved players that reach the senior NT, combined with a high turnover of the other players would in these analyses be a more collectivistic approach. Although this should probably be considered an individualistic approach. More studies using person-centered approaches to look at players’ pathways would provide more detailed views of selection strategies.

## Conclusion

We can highlight four findings from this study of selection strategies in European basketball NT programs. First, there are considerable differences in selection strategies between European basketball youth NT programs, with a difference of eight players per generation between countries that select the highest and lowest number of youth players. Second, NT programs that select a higher number of youth players seem to perform better, both at the youth and senior level. Third, differences in the number of licensed basketball players explain part of the difference in selection strategy between countries; it explains almost none of the relationship between selection strategy and success on the men’s side but a small part of the relationship on the women’s side. Fourth, within countries, generations with a higher number of selected youth NT players produce a higher number of senior NT players.

## Data Availability Statement

The original contributions presented in the study are publicly available. This data can be found here: https://doi.org/10.17605/OSF.IO/ZHVDS.

## Author Contributions

AK, AP-F, and EL conceptualized the study. AK collected and analyzed the data. AK and AP-C wrote the original manuscript draft. AP-F, AP-C, EL, and ER reviewed and edited the manuscript. EL and ER supervised the work. All authors contributed to the article and approved the submitted version.

## Conflict of Interest

AK worked as a youth national team assistant coach for Sweden during some of the seasons included in the current study. The Swedish Basketball Federation was not involved in any way in the conception of the study, nor in the collection, analysis, and interpretation of the data and results. The remaining authors declare that the research was conducted in the absence of any commercial or financial relationships that could be construed as a potential conflict of interest.
