# Supplementary material for: Talent Selection Strategies and Relationship With Success in European Basketball National Team Programs
Source: Front Psychol. 2021 Jun 11;12:666839. doi: 10.3389/fpsyg.2021.666839 (PMC8232224; doi:10.3389/fpsyg.2021.666839)
Supplement: Supplementary file 1 [file Data_Sheet_1.PDF]

Supplementary Material

Supplementary Table 1. Descriptive statistics of countries on women's side.

Country	Senior ranking	Youth Ranking	Licensed Players	Nr Gen	Avg nr youth	CV% nr youth	Avg nr senior
Belarus	475.1	29	2 362	12	9.3	33.3	1.4
Belgium	585.7	160	21 632	12	12.1	16.0	2.6
Bosnia and Herzegovina	149.2	18	794	9	8.6	29.9	3.0
Bulgaria	124.9	0	738	12	11.8	26.4	1.8
Croatia	233.7	76	7 210	12	12.8	18.4	1.6
Czech Republic	334	211	17 456	12	14.3	20.7	1.4
Denmark	111.8	13	5 107	8	13.0	10.1	0.3
Estonia	106.2	3	733	7	8.9	21.1	3.0
Finland	97.5	9	900	12	11.8	16.8	2.0
France	640.8	414	332 555	12	15.1	17.3	1.4
Germany	149.8	151	79 986	12	13.8	18.7	2.8
Great Britain	333.5	3	9 491	12	13.7	25.6	2.3
Greece	368.0	10	41 979	12	12.5	23.3	1.0
Hungary	257.9	247	10 460	12	13.9	28.7	1.9
Ireland	91.9	5	5 336	6	15.2	31.6	2.7
Israel	147.6	20	4 291	12	12.0	15.5	1.0
Italy	345.4	315	49 279	12	14.8	16.9	1.9
Latvia	303.4	141	1 600	12	11.1	16.5	1.8
Lithuania	233.1	103	2 337	12	12.3	17.8	2.3
Netherlands	136.5	57	13 133	12	11.7	18.4	3.4
Poland	167.9	49	9 173	12	13.9	21.5	2.3
Portugal	130.9	56	1 670	12	11.2	20.5	2.8
Romania	138.1	3	7 575	12	11.8	18.9	1.8
Russia	400.9	288	3 985	12	15.6	13.0	1.6
Serbia	579.4	108	2 242	9	12.1	19.6	1.6
Slovakia	275.2	17	2 278	12	12.4	25.6	2.5
Slovenia	232.6	38	425	10	9.7	28.4	2.3
Spain	692.2	473	94 698	12	13.9	12.4	1.6
Sweden	315.0	22	10 500	12	14.4	17.1	2.8
Switzerland	98.1	0	3 425	5	11.0	6.4	2.5
Turkey	598.7	67	9 525	12	11.8	16.4	2.5
Ukraine	270.5	0	1 022	12	12.0	19.5	2.0

Supplementary Table 2. Descriptive statistics of countries on men's side.

Country	Senior ranking	Youth Ranking	Licensed Players	Nr Gen	Avg nr youth	CV% nr youth	Avg nr senior
Austria	172.6	0	13 920	10	14.5	13.5	2.3
Belarus	195.8	0	4 930	9	12.8	12.2	1.0
Belgium	281.9	0	61 809	12	15.6	11.1	2.8
Bosnia and Herzegovina	226.7	110	4 034	8	15.6	12.3	1.7
Bulgaria	204.6	0	1 222	12	14.1	11.5	2.5
Croatia	522.4	220	12 780	12	17.1	11.0	1.3
Czech Republic	591.5	19	28 007	12	15.3	12.9	1.8
Denmark	164.5	0	11 896	8	13.6	13.6	3.2
Estonia	209.9	25	2 635	12	13.5	15.6	2.0
Finland	319.4	75	540	12	15.3	16.3	1.4
France	656.5	367	546 632	12	19.9	16.2	2.6
Germany	495.7	112	203 439	12	18.0	14.6	3.3
Great Britain	242.7	23	21 450	12	17.8	11.2	2.0
Greece	647.4	158	189 336	12	14.9	20.9	1.4
Hungary	262.2	0	26 360	12	17.1	14.2	1.8
Iceland	218.2	0	4 766	5	11.2	29.2	2.0
Ireland	90.0	0	11 692	6	17.0	13.9	0.0
Israel	245.7	33	22 359	12	16.2	9.8	1.6
Italy	577.9	202	109 862	12	18.8	15.5	2.4
Latvia	382.2	112	4 175	12	15.3	16.5	2.6
Lithuania	635.2	339	8 396	12	18.4	11.2	1.9
Luxembourg	110.9	0	2 530	10	12.4	12.1	3.0
Montenegro	391.4	103	2 268	9	15.0	13.7	1.2
Netherlands	231.2	8	29 348	12	17.1	13.8	2.6
North Macedonia	187.6	7	2 239	8	13.9	9.0	1.8
Norway	104.2	0	7 195	5	13.0	22.4	0.0
Poland	567.6	4	16 786	12	17.3	20.0	1.9
Portugal	153.0	0	3 203	12	14.1	14.0	2.4
Romania	179.4	0	11 610	12	14.3	17.4	1.4
Russia	585.4	105	7 455	12	19.7	17.9	2.0
Serbia	661.3	309	16 766	9	16.7	17.5	2.8
Slovakia	134.0	4	2 348	12	15.2	19.8	2.9
Slovenia	496.4	67	2 116	12	15.1	14.0	2.3
Spain	722.9	378	276 273	12	16.7	10.7	1.8
Sweden	176.4	15	11 700	12	16.9	13.0	2.5
Switzerland	151.5	0	9 360	9	15.3	25.7	2.6
Turkey	513.2	311	42 813	12	14.5	16.5	2.4
Ukraine	353.0	15	3 136	12	15.5	10.8	2.5
